# Supplementary material for: Quantifying molecular bias in DNA data storage
Source: Nat Commun. 2020 Jun 29;11:3264. doi: 10.1038/s41467-020-16958-3 (PMC7324401; doi:10.1038/s41467-020-16958-3)
Supplement: Supplementary file 1 — Supplementary Information [file 41467_2020_16958_MOESM1_ESM.pdf]

Quantifying Molecular Bias in DNA Data Storage

Supplemental Material

Chen *et al*.

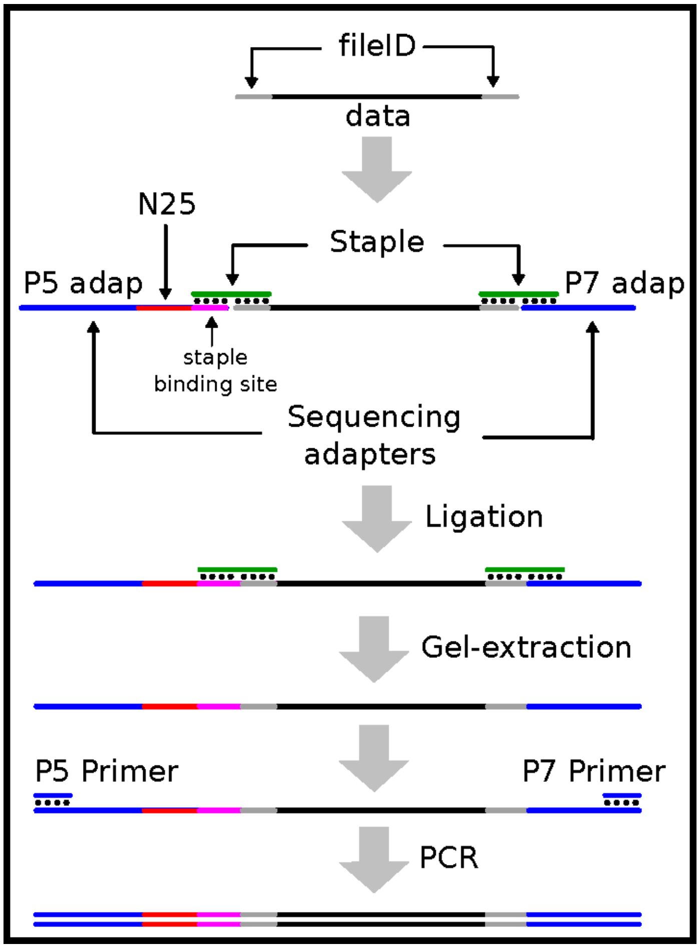

Supplementary Figure 1. DNA data strands are assembled with sequencing adapters using two staples. Note that one of the adapters contains a randomized region (N25) which serves as UMI. After assembly, DNA nicks are sealed using T4 DNA ligase. A denaturing PAGE (D-PAGE) gel is then used to extract the ligated strands. Finally, two end primers are used to enrich the full-length product for sequencing on an Illumina instrument.

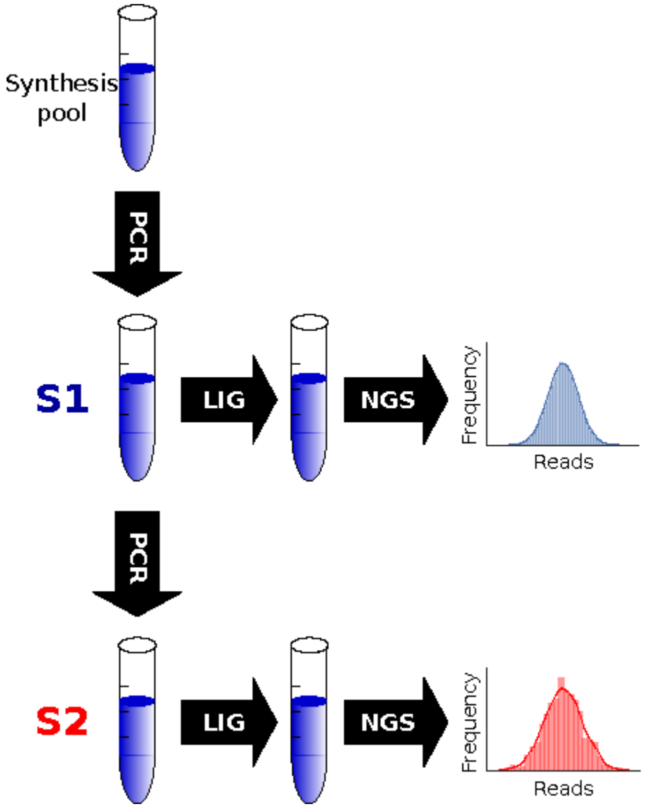

Supplementary Figure 2. To quantify PCR bias, we used a 2-step PCR-sequencing method. The synthesis pool was PCR-amplified with a pair of primers to obtain an amplified file *S1*. *S1* was PCR-amplified again with the same primers to obtain *S2*. *S1* and *S2* were ligated to Illumina sequencing adapters and sequenced to get their oligo copy distributions. The S1 distribution informs the distribution before PCR, while the S2 distribution informs the distribution after PCR. By comparing the distributions of *S1* and *S2*, PCR bias can be quantified. The sample size (number of unique sequences) in Fig. 4c and Fig. 4d is 1,536,168 and 1,358,998, respectively.

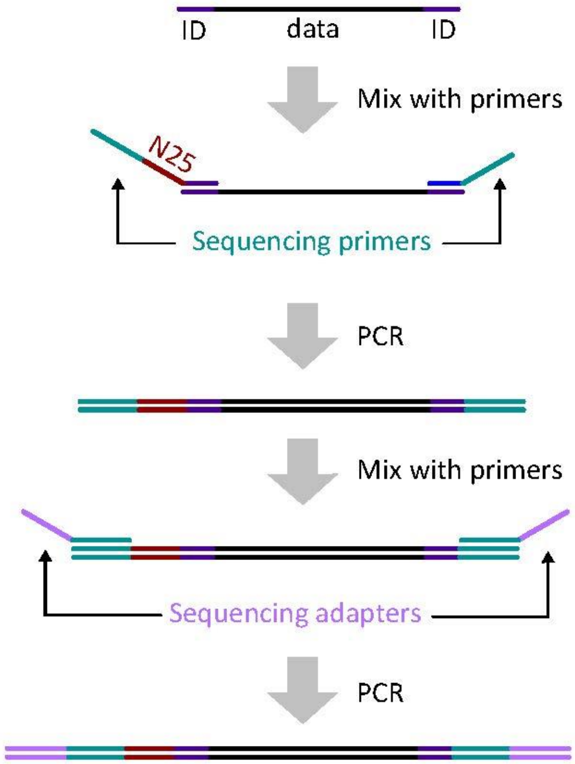

Supplementary Figure 3. Workflow of dilution-PCR experiments. A DNA pool is first amplified with primers that include Illumina sequencing primers overhangs and a randomized region (N25). The randomized region is used for increasing the diversity for an Illumina NextSeq instrument. After that, the amplified oligos were amplified with another set of primers with Illumina sequencing adapters.

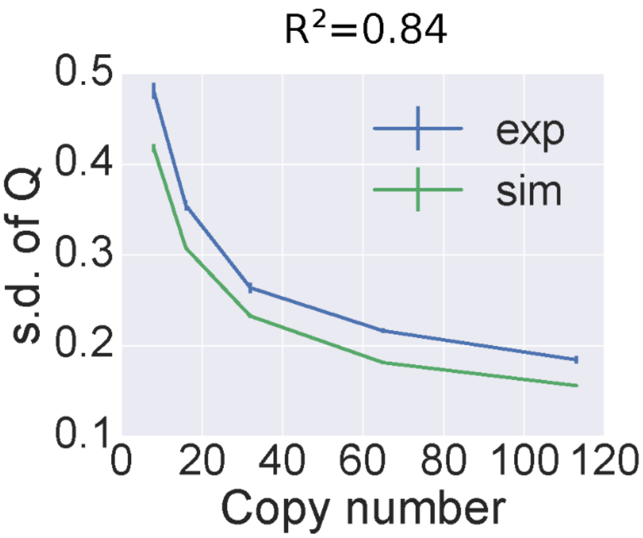

Supplementary Figure 4. Standard deviation of *population fraction change Q* of the post-PCR DNA versus average copy number of the pre-PCR mix. The blue trace shows the standard deviation of Q in the post-PCR experimental data sequenced and then sampled at 200x coverage where the pre-PCR mix contains an average of 8 to 113 copies per sequence and its Q is calculated comparing to the same mix at average 200 copies per sequence. The green trace shows the simulated data, i.e., the standard deviation of Q in the post-PCR mix after simulated sequencing and sampling at 200x coverage where the pre-PCR mix contains an average of 8 to 113 copies per sequence and its Q is calculated comparing to the same mix at average 200 copies per sequence with c.v. = 0.32. The model prediction (green) captures a trend similar to the experimental data (blue) with R^2=0.84. The error bars of the experimental data indicate standard error calculated from triplicate experiments. The simulation is plotted in green color, and the error bars indicate standard error from 100 repeated simulations.

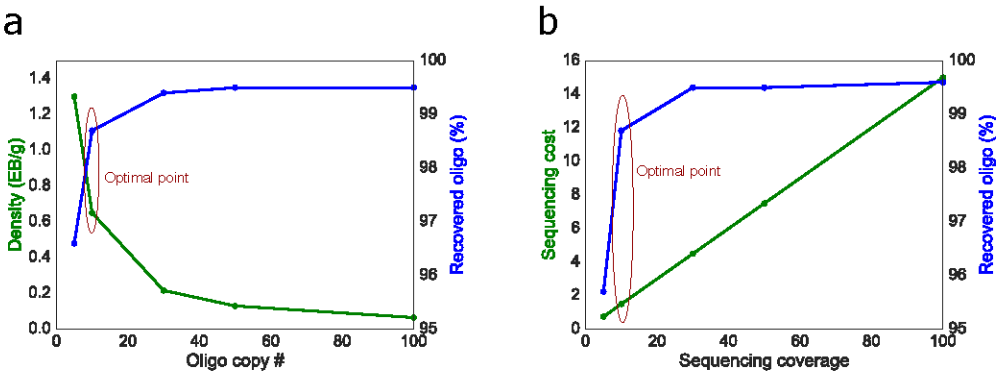

Supplementary Figure 5. Examples of physical redundancy and sequencing redundancy optimization curves. A synthesis pool was generated with N_{seq} = 10,000 total number of sequences, with normally distributed copy numbers with a mean of $\bar{n}_{syn}$ = 10^8 and standard deviation $\sigma = 3.2 * 10^7$. **a** The x-axis represents average copy number of the pool stored. PCR simulation was performed with 20 cycles of PCR amplification with *P*=0.95, and high throughput sequencing with average sequencing coverage $\bar{n}_r$=10. The stored pool density is shown in green, and the percentage of recovered oligos (with at least one read) is shown in blue. As shown in the figure, storing more copies will result in more oligos recovered from sequencing, but this affects physical density negatively. From this example, the optimal point for oligo copy number is around 10 copies for a good tradeoff between physical density and percentage of recovered oligos. **b** The pool was simulated to store an average copy number $\bar{n}_0$ =100, followed by 20 cycles of PCR amplification with *P*=0.95, and high throughput sequencing with average sequencing coverage $\bar{n}_r$ shown in the x axis. The assumed sequencing cost is $100 per Gbases in this example. As shown in this example, increasing the sequencing coverage can increase the percentage of recovered oligos initially, but the improvement quickly saturates beyond an average of 20 sequencing reads. In this example, the optimal point for sequencing coverage is around 10 for a good tradeoff between cost and the percentage of recovered oligos.

Name	Sequences	Length (bp)
P5 adapter	AATGATACGGCGACCACCGAGATCTACACTCTTTCCCTACACGACGCTCTTCCGATCTNNNNNNNNNNNNNNNNNNNNNNNNNAGTGAGGTAGAGGTGTATTC	103
P7 adapter	GATCGGAAGAGCACACGTCTGAACTCCAGTCACATCACGATCTCGTATGCCGTCTTCTGCTTG	63
P5 staple	TGCTGGTAACAACTTGCCTTGAATACACCTCTACCTCACT/3SpC3/	40
P7 staple	AGACGTGTGCTCTTCCGATCTTGGTTTGATTACGGTCGCA/3SpC3/	40
P5 primer	AATGATACGGCGACCACCGAGA	22
P7 primer	CAAGCAGAAGACGGCATACGAG	22

Supplementary Table 1. Sequences of UMI labeling. /3SpC3/ represents a C3 spacer modification at the 3'end.

Pool	File	Forward Primer	Reverse Primer	File Size (bytes)	Number of sequences
Ready-to-sequence pool		AATGATACGGCGACCACCGAGA	CAAGCAGAAGACGGCATACGAG	10,686,220	1,536,168
Homopolymer Pool	1	TCCTGCTTGCGTTAAATGGA	TTCCGCAAGACTTATTGGCA	5,043,000	241,648
Homopolymer Pool	2	ACCGCGCTCGAAGAATTTAA	TCGCAACACCTTTCGTACAA	6,295,900	301,680
Homopolymer Pool	3	AAACAAAGTTAGCGGCTCGT	AGGCCGCGAATTTGGATTAT	5,815,000	278,640
Homopolymer Pool	4	AATTTGGCATTCACCGTGGA	AGTCGCCAAATAAGTGCCAT	6,690,000	320,565
Homopolymer Pool	5	AGCCTTGTGTCCATCAATCC	ATTAGCCAAACCATAGCGCA	600,200	28,761
Homopolymer Pool	6	TGTATTTCCTTCGGTGCTCC	AAACCAGACCGTTGTCGAAA	573,700	27,491
Homopolymer Pool	7	TGTGTTCCTCCTCGGTATGA	AGGAAGCGCCAACTAATTGT	180,500	8,650
Homopolymer Pool	8	TAGCCTCCAGAATGAAACGG	TACACACGGTTTGGCTTGAA	3,163,000	151,563
Serial dilution PCR experiment (Fig. 5)		ACATTCCGTGCCATTGGATT	TTTGTGGAACGATTTGCCGA	115,394	7,373

Supplementary Table 2. Pools/files used in this study. All encoded pools/files are listed. All files were encoded in 150-base DNA strands.
